# HIV-1 Diversity in the Envelope Glycoproteins: Implications for Viral Entry Inhibition

**DOI:** 10.3390/v5020595

**Published:** 2013-02-06

**Authors:** Leonardo Augusto Luvison Araújo, Sabrina E. M. Almeida

**Affiliations:** Centro de Desenvolvimento Científico e Tecnológico (CDCT), Fundação Estadual de Produção e Pesquisa em Saúde (FEPPS), Porto Alegre, 90610-000, Brazil.

**Keywords:** HIV-1, entry inhibitors, envelope, diversity

## Abstract

Entry of HIV-1 into a host cell is a multi-step process, with the viral envelope gp120 and gp41 acting sequentially to mediate the viral attachment, CD4 binding, coreceptor binding, and fusion of the viral and host membranes. The emerging class of antiretroviral agents, collectively known as entry inhibitors, interfere in some of these steps. However, viral diversity has implications for possible differential responses to entry inhibitors, since envelope is the most variable of all HIV genes. Different HIV genetic forms carry in their genomes genetic signatures and polymorphisms that could alter the structure of viral proteins which are targeted by drugs, thus impairing antiretroviral binding and efficacy. This review will examine current research that describes subtype differences in envelope at the genetic level and the effects of mutations on the efficacy of current entry inhibitors.

## 1. Introduction

Human immunodeficiency virus (HIV) is the etiologic agent of acquired immuno-deficiency syndrome (AIDS), one of the most devastating infectious diseases to have emerged in recent history. The origin of pandemic form of HIV-1, the main (M) group, has been traced to a simian immunodeficiency virus (SIV), which was probably passed from chimpanzees to human hunters through bloodborne transmission [1]. HIV-1 evolves quickly due a rapid reproductive rate and reverse transcriptase is error prone. Thus several intrinsic mechanisms as well as dissemination involved a number of population bottlenecks ensure rapid viral evolution leading to the predominance of different group M lineages around the world. HIV-1 group M has been currently divided into lineages or subtypes A, B, C, D, F, G, H, J, and K as well as circulating recombinant forms (CRFs) and unique recombinant forms (URFs) [1,2]. Genetic variation within a subtype can be 15 to 20%, whereas variation between subtypes is usually 25% to 35%. Differential characteristics of viral subtypes and their interactions with the human host may influence HIV transmission and antiretroviral therapy. The most striking changes in diversity are in the envelope glycoproteins (Env) gp41 and gp120, which are associated with viral transmission [3] and host cell tropism [4]. The gp41 plays a crucial role in the depletion of CD4+ T cells by inducing the death of cell. HIV-1 envelope glycoprotein-mediated entry and fusion have been a target for the development of antiretrovirals, known as entry inhibitors [5]. This review will examine current research that describes subtype differences in envelope at the genetic level and the effects of resistance mutations on the efficacy of current entry inhibitors.

## 2. HIV Entry and Its Inhibition

**Figure 1:**
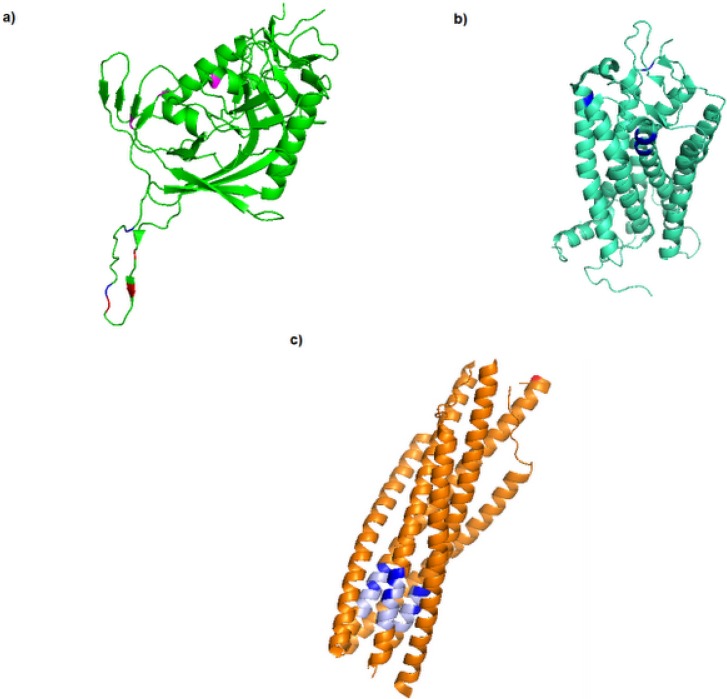
Ribbon diagram of gp120 (pdb 2QAD), bovine rhodopsin (pdb 1F88A) and SIV gp41 (pdb 1QBZ). (**a**) Ribbon diagram of gp120 (shown in green) bound to CD4 and Fab 17b (not show). The sites related to vicriviroc (305, 308, 315), maraviroc (316, 323) and BMS-806 (426, 434, 475) resistance is shown (in red, blue and magenta, respectively). (**b**) Structure of bovine rhodopsin, used as template in homology modeling procedure to CCR5. All CCR5 inhibitors are believed to occupy a binding pocket formed at the base of the extra cellular loops of the CCR5 receptor (shown in blue). (**c**) gp41 enfuvirtide bind site and resistance mutations (36, 37, 38, 40, 42, 43, 89). Residues in light blue correspond to both the enfuvirtide binding site and the resistance mutations.

Significant progress has been made in understanding HIV-1 envelope structure and the process of entry into a host cell. The envelope gene encodes a protein precursor gp160, which undergoes maturation steps in the endoplasmic reticulum yielding the surface unit gp120 and the transmembrane region gp41 [6]. For entry of HIV-1 into a target cell, the gp120 subunit of the viral envelope glycoprotein associates with the CD4 receptor and the CCR5 coreceptor, inducing a series of conformational changes in Env that culminate in virus and host cell membrane fusion. Most primary HIV-1 strains use the chemokine receptor CCR5 as coreceptor in conjunction with CD4 for virus entry; however, some strains evolve to use a related receptor, CXCR4, either in place or in addition to CCR5 [7]. The coreceptor binding triggers the gp41 N-terminal heptad region (HR1 or NHR) and the C-terminal heptad region (HR2 or CHR) exposure, forming a triple-stranded coiled-coil that approaches the host and viral membranes, forcing the fusion peptides into the target cell membrane. The gp120-gp41 complex undergoes a series of conformational changes during the entry process. Knowledge of these steps in order to design entry inhibitors is very important, since rational drug discovery is based on an understanding at the molecular level of the process to be inhibited [8]. Nevertheless, many details are missing and many challenges remain in achieving this goal. There are several possible targets for the development of drugs with synergistic effects in inhibiting viral entry steps at which interference with the process can be attempted. Generally, these targets can affect viral entry by inhibition of CD4 binding, inhibition of coreceptor binding (CCR5 antagonists), and blocking of the gp41 conformational changes that permit viral fusion (Fusion inhibitors) (Figure 1). For more details, see the review [9]. To the best of our knowledge, only two entry inhibitors have been approved by the Food and Drug Administration (FDA): enfuvirtide (T20), a fusion inhibitor, and selzentry (maraviroc), a CCR5 antagonist. One additional entry inhibitor in an advanced stage of clinical development is the CCR5 antagonist vicriviroc (SCH 417690); however, due to results obtained in a recent phase III trial development will be discontinued.

## 3. Diversity in the Envelope Glycoproteins

**Figure 2:**
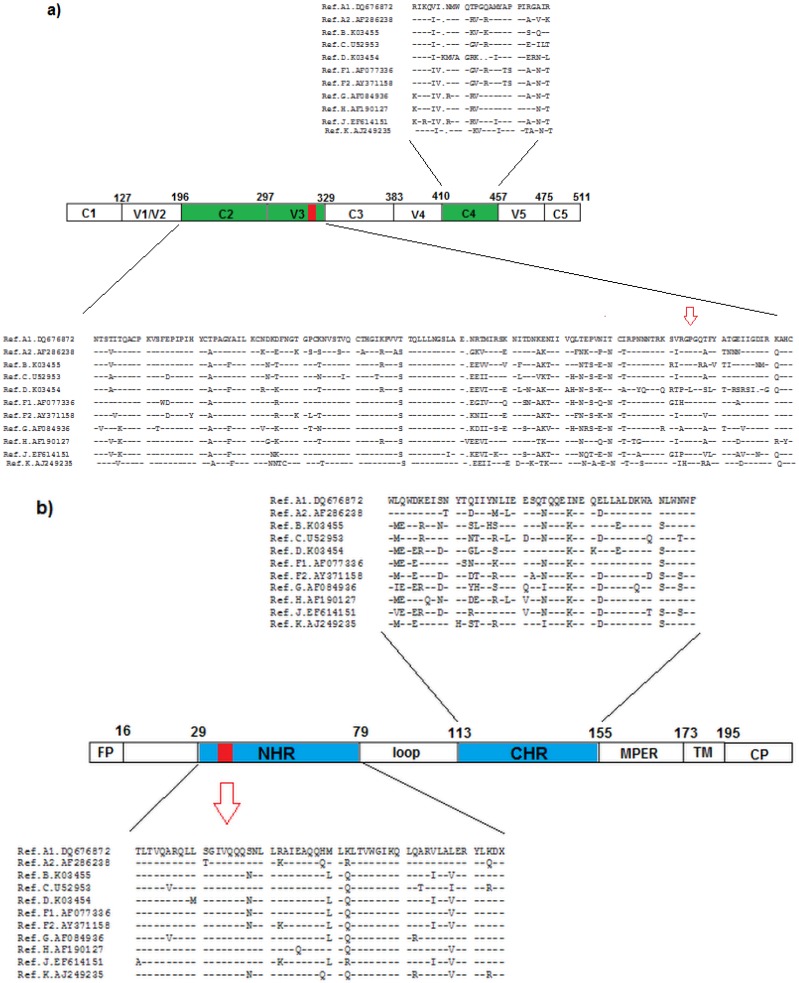
Schematic view of the HIV-1 HXB2 gp120 and gp41 molecules. Boxes designate crucial regions involved in resistance to entry inhibitors. The sequences contain representative alignment of each HIV-1 group M subtype (obtained in Los Alamos HIV database). (**a**) The constant (C1, C2, C3, C4, C5) and variable regions (V1,V2, V3, V4, V5) of gp120. Changes in gp120 C2, V3 and C4 are related to resistance to the CCR5 antagonist and CD4-gp120 inhibitor. The arrow points to the end of the V3 loop where the resistance mutations to CCR5 agonists are located (**b**) Schematic diagram of HIV-1 gp41. FP, fusion peptide; NHR, N-terminal heptad repeat; CHR, C-terminal heptad repeat; MPER, membrane-proximal external region; TM, transmembrane domain of gp41; CP, cytoplasmic domain. The fusion inhibitor enfuvirtide targets the GIV motif in the NHR. The mutations leading to resistance to enfuvirtide are located between residues 36-45 in the NHR region of gp41 (red band and arrow). Resistance mutations in the CHR region also have been detected.

As stated earlier, group M is responsible for the current AIDS pandemic and exhibits exceedingly high levels of viral genetic diversity. The subtypes of group M can differ by around 35% in the envelope glycoproteins of the virus (Figure 2). The pattern of diversity and adaptive evolution in the Env gene was examined by several studies, as in Choisy *et al.* and Travers *et al.,* that used multiple subtypes to identify sites evolving under positive selection in gp120 and gp41 [10,11]. A large number of amino acid sites are evolving under positive selection in HIV-1 group M envelope protein. When the selection pressure is compared by subtype, several sites are under positive pressure in some subtypes and under negative pressure in others. The presence of such sites indicates unique selective pressures for particular subtypes, which may lead to different phenotypic characteristics within HIV-1 group M evolution and account for the various levels of fitness. Insertion and deletion events occur throughout Env and are maintained through positive selection, particularly within the hypervariable loops, which acquire significant length variation [12,13].

The tip of the V3 loop, which is a target for antibody neutralization and plays a role in the tropism and infectivity of the virus, seems to be under selection pressure for length as it is almost always 35 residues long [14,15]. Generally, CXCR4-using viruses carry positively charged amino acids at positions 11 and/or 25 in the V3 loop, while CCR5-tropic viruses do not. The tip contains a highly conserved motif, Gly-Pro-Gly-Arg/Gln (GPGR/Q, residues 312–315 in the HXB2 numbering), usually GPGQ among all HIV-1 subtypes, whereas GPGR predominates in the B subtype. The variability and the ratio of non-synonymous (*dN*) to synonymous (*dS*) in a comparison using subtype B and C V3 sequences deposited in the Los Alamos HIV Database exhibits higher entropy and dN/dS ratio in subtype B [16]. Interestingly, the flanking regions of V3 in subtype C exhibits higher entropy and dN/dS ratio than subtype B. These findings suggest that the adaptive pressures that have shaped Env in each lineage are distinct, and this may have formed the basis for conformational differences between subtypes [17,18]. The selective pressure exerted either by CTL or neutralizing antibodies can account for particular evolutionary patterns in the Env gene. Although genetic diversity of the Env has been extensively studied, less information is available concerning functional diversity of these proteins. Studies have focused on subtype-specific conformational differences of the V3 and C3 regions. These studies demonstrate that there are intrinsic differences within the V3 stem and turn region (positions 9 to 24) in entropy and C3 region in entropy and amphipathicity between subtypes B and C [15,19]. Subtype-specific patterns of sequence polymorphism in gp41 HR1 and HR2 regions have also been shown, suggesting that selection pressures could differ between subtypes [20,21,22,23]. These studies mainly concentrated on the comparison of subtypes B and C point to possible structural differences, which can give different responses to entry inhibitors. Further studies are needed to compare other subtypes to elucidate major structural differences, and to determine how these affect the activity of entry inhibitors.

## 4. Resistance to Entry Inhibitors

Certain mutations impacting drugs in the entry inhibitor have been identified *in vitro* passage experiments, examination of clinical isolates and correlation studies between genotype at baseline and virologic response in patients exposed to the drug [24,25]. The most common genetic route to CCR5 inhibitor resistance involves multiple sequence changes in V3 and result in gaining the ability to enter cells using the inhibitor-CCR5 complex while retaining the use of free CCR5 [26]. A rare pathway of HIV-1 resistance to small molecule CCR5 inhibitors such as vicriviroc involves changes solely in the gp41 fusion peptide [27]. These data should be interpreted in light of the fact that subtype B viruses are most frequently used in biological studies of resistance to entry inhibitors. The information on non-B subtypes resistance remains very limited. Araújo *et al.* and Gonzales *et al.* showed a high prevalence of resistance mutations for maraviroc and vicriviroc in HIV-1 subtype C, which may suggest a limited efficacy of CCR5 inhibitors in this subtype [28,29]. Natural gp120 variability among different HIV-1 subtypes may account for differences in baseline susceptibility to entry inhibitors. This is the case for subtype C and recombinant subtype AE (CRF01_AE) resistance to CD4–gp120 binding inhibitors, which seem to be naturally resistant to BMS-806 [30].

Studies using enfuvirtide, a fusion inhibitor, showed that differences in the susceptibility of enfuvirtide-naive virus and the development of resistance are associated with changes in a conserved amino acid triad (GIV) at positions 36–38 in the NHR region of gp41 (Figure 2). Mutations in the CHR region also have been detected in enfuvirtide-resistant HIV-1 variants that emerge under the selective pressure of enfuvirtide [31,32]. When analyzing the evolution of Env sequences, enfuvirtide susceptibility, and Env replicative capacity, the epistasis appears to play a critical role in the selection of NHR mutations and the expression of enfuvirtide resistance, altering the evolution of HIV-1 under fusion inhibitor selective pressure [33,34]. The viral envelopes with high-affinity binding to the coreceptor fused more quickly than viral envelopes with lower affinity, reducing the kinetic window during which the viral envelope is sensitive to enfuvirtide [35]. These findings emphasize the complexity involved in the emergence of viral susceptibility to fusion inhibitors, and suggest that the development of resistance can be affected by viral replicative capacity, tropism and coreceptor affinity [36,37,38]. Since the epistatic effects, tropism and coreceptor affinity differ among subtypes, the resistance may be influenced by the evolutionary history of the HIV strain. For example, there was no evidence for baseline resistance to enfuvirtide in subtype C viruses despite significant differences relative to subtype B in gp41 [39]. In contrast, Yu *et al.* demonstrates that there were significant differences in baseline susceptibility to HIV entry inhibitors among B’ isolates (also known as Thai B), CRF07_BC and CRF01_AE [40]. Since the majority of HIV-1 infections worldwide involve non-subtype B subtypes and genetic diversity is increasing, additional studies of baseline resistance to entry inhibitors against non-subtype B viruses will be important from a global perspective.

In summary, differences in amino acid composition between HIV-1 clades can lead to differences in susceptibility to ARV drugs. Susceptibility of non-B subtypes to ARV drugs has been less well studied than subtype B mainly because of the predominance of subtype B in developed countries. Therefore, the extreme genetic diversity in envelope of HIV-1 poses a significant challenge for entry inhibitors design and rational of optimal therapeutic regimens to treat patients. Studies that continue to uncover subtype-specific differences in Env function and structure will be necessary both for discovering new inhibitors and to improving the therapeutic applications for entry inhibitors.
